# Functional Assessment and Injury Risk in a Professional Soccer Team

**DOI:** 10.3390/sports5010009

**Published:** 2017-01-22

**Authors:** Pedro Gómez-Piqueras, Sixto González-Víllora, María del Pilar Sainz de Baranda Andújar, Onofre R. Contreras-Jordán

**Affiliations:** 1 Faculty of Education, University of Castilla-la Mancha, 02071 Albacete, Spain; Onofre.Cjordan@uclm.es; 2 Faculty of Education, University of Castilla-la Mancha, 16071 Cuenca, Spain; Sixto.Gonzalez@uclm.es; 3 Faculty of Sport Sciences, University of Murcia, 30720 San Javier, Spain; Psainzdebaranda@um.es

**Keywords:** functional condition, suffered injury, football, sport performance

## Abstract

At the last World Conference on Sport and Physical Therapy celebrated in Bern (Switzerland, 2015), it was confirmed that the functional skills of an athlete are a very important variable to be considered in the recovery of an injury. On the other hand, its use as a predictive risk tool still lacks solid evidence. The purpose of this study was to determine whether a battery of functional tests (FPT) could be used as a preliminary measure for the season in order to identify the injury risk in a professional soccer team in the Spanish Second Division B League. Fifty-two soccer players (ages of 25.3 ± 4.6 years, 10.33% ± 0.9% fat) were functionally assessed during two seasons (2012–2013 and 2013–2014) and analyzed from an injury perspective. A total of 125 injuries were recorded. The sample was grouped based on the number of injuries and the required absence days. Except for the bipodal vertical jump (CMJ), none of the functional tests revealed differences among the groups. The correlation study between the functional condition and the suffered injuries did not show any significant results.

## 1. Introduction

The negative consequences that all injuries cause have become one of the main questions pending a solution by the doctor/technician staff of professional sports clubs [1]. Their multidimensional origin [2], the product of the interaction of the context jointly with a series of intrinsic and extrinsic conditioning factors [3], hinders the detection of the factors which entail the highest risk for an athlete [4,5,6].

The most outstanding extrinsic factors are the type of activity and the specific sports gesture, the dynamic training load, the exposure time to training sessions and competition, the material and equipment, the type of surface and the sports context [7,8]. Likewise, as intrinsic factors to be taken into account to prevent injuries, we detected areas such as the existence of a previous injury [9,10], age [11,12], body composition [13,14], psychological skills management such as stress, anxiety, emotional mood or personality [15], and a poor physical condition level [16].

The athlete’s functional skills, as confirmed at the last World Conference on Sport and Physical Therapy celebrated in Bern (Switzerland), is another one of the intrinsic variables to be highlighted in a preferential way in the prevention and re-adaptation of injuries [17]. For the measurement of these types of skills, functional tests (FPT) have been used for quite some time; however, still without solid evidence [18]. These measurement tools globally integrate the subject and closely simulate the conditions of a specific sport or activity [19], and can thus simultaneously measure abilities such as flexibility, strength, endurance, coordination, balance and motor control [20].

Although the importance given to the FPT by sports managers is high [21], their use is not as frequent as it should be [22]. One of the reasons is that the predictive values of these types of tests have not shown sufficient evidence until now [23]. In this search for evidence, and with inconclusive results, this type of test has been used to predict the injury of a lower limb [24,25], and to identify the imbalances related to the ankle [26] and the knee [27,28]. Specifying the soccer player’s risk of injury during the preseason with the use of inexpensive measures, which require little time and a low risk, would reduce the number of injuries and their associated economic-sports cost [24].

The purpose of this prospective study was to determine if a battery of functional tests could be used as a preliminary measure prior to the season in order to identify the injury risk in a professional soccer team in the Spanish Second Division B League. The initial hypothesis was that the soccer players with the worst scores in the functional tests would suffer a higher number of injuries throughout the season.

## 2. Materials and Methods

### 2.1. Participants

Fifty-two healthy male soccer players, who belong to a soccer team in the Spanish Second Division B League, with an average age of 25.3 ± 4.6 years, a height of 177.8 ± 7.2 cm, a body mass of 76.6 ± 6.6 kg, a fat percentage of 10.33% ± 0.9% and prior experience in the sport modality for at least eight years, were functionally assessed during two consecutive seasons (2012–2013 and 2013–2014) and analyzed from an injury perspective. The players who were injured performed the same test at other moment of the season.

The training methodology to which these players were subjected throughout the two seasons had a marked integrated nature. Approximately 80% of the time was devoted to high specificity tasks and 20% to analytical tasks with a preventive and complementary nature. On average, each of the two seasons was composed of 238.7 ± 2.2 training sessions lasting approximately 80 min and 43.2 ± 1.2 official matches between league, king’s cup and playoffs.

The study was conducted following the ethical recommendations from the Declaration of Helsinki and Ethics Committee of the University of Castilla La Mancha, where all the participants were informed of the study’s characteristics and provided their consent to participate.

### 2.2. Procedure

During two seasons, all the sports injuries, understood as “any physical harm suffered by a player resulting from a soccer game or training session, independently of the necessity for medical care or the time of absence from the soccer activities” [29] were recorded during training and matches. In addition, the variables recommended by the literature which refer to epidemiology (type, location and severity) [29] were described and registered by the heads of the medical staff (physiotherapist and doctor) using the nomenclatures and categories agreed upon in this consensus document [29].

All the soccer players were assessed using a battery of six functional tests proposed by a team of experts in injury recovery. To avoid the limited predictive value of a single functional measurement during the season [30], we chose to carry out three measurements: July (preseason), December (middle season) and June (final season). The mean value of the three measurements was recorded. Those players who belong to the team during the two seasons, obtained a total of six measurements (three per season). In this case, the mean value was also recorded.

The testing was guided by two evaluators with extensive experience in the field (University graduates in Sport Sciences and specialists in injury prevention) and familiar with the use of the selected tests. All the players were assessed in the team’s usual facilities, with the usual work-out clothing and the subjects had not carried out significant efforts in the 48 h before the measurement. To minimize the external atmospheric effects, all tests were performed on an artificial surface under cover. Previously an initial awareness session was carried out where the athletes learned about the functioning of each of the tests and their proper execution. On subsequent days, the players were summoned in groups of five in a single session. Each player was instructed and motivated to provide their maximum effort in each of the tests, where they could withdraw from the study at any time.

After the players were instructed to maximize their performance and recall the functioning of the tests, the assessment began with a standard warm-up. This warm-up consisted of 5 min of a continuous running at low intensity and 5 min of articular mobility exercises with running sessions of progressive intensity concluding in a sprint. To minimize fatigue between test and test, a rest period of 15 min was established between tests.

### 2.3. Functional Tests

**Figure 1:**
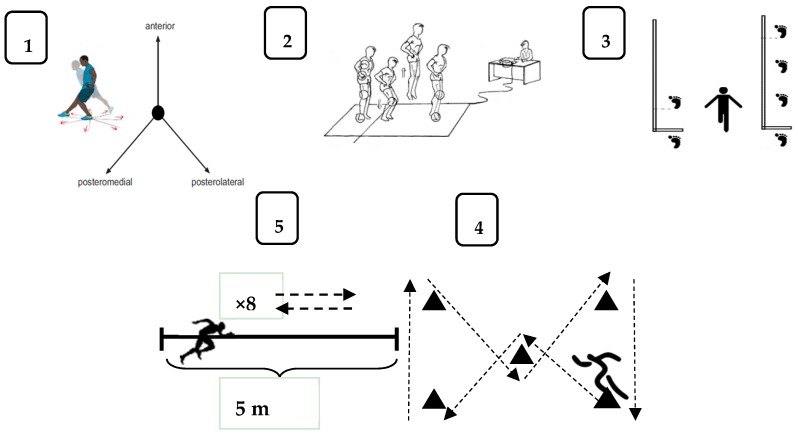
Functional tests: (**1**) Y balance; (**2**) CMJ; (**3**) single hop and triple hop; (**4**) barrow test; (**5**) shuttle Run 8 × 5 m.

By order of execution, the functional tests were: Y balance test [31], counter movement jump (CMJ) [32], single hop (SLHT) and triple hop (THT) [33], barrow test [34] and 8 × 5 m shuttle run sprint test [35] (Figure 1).

To facilitate the reproducibility of this study, the protocols used in each of the tests were as follows.

### 2.4. Y Balance Test/Posture Control

The Y balance test [31] is presented as a simple and inexpensive tool with acceptable levels of reliability and validity [36]. The original test consists in tracing an asterisk on the ground with eight lines which each intersect each other at a 45° angle. In the abbreviated version, the Y balance Test only measures three of the eight directions (anterior, posteromedial and posterolateral). The subject, in a unipodal support on the leg to be assessed, is placed in the center of the asterisk, mobilizing the contralateral leg in the direction of the three marked directions, attempting to achieve the maximum distance possible in each of them. This position requires ankle dorsiflexion, knee flexion, and hip flexion as well as suitable values of strength, proprioception and neuromuscular control [37]. The subject, barefoot, to eliminate shoe adjustments [38], in shorts and with the hands on the hips, tried with the most distal part of the contralateral foot to reach the maximum distance in each of the three directions [39].

### 2.5. CMJ/Bipodal Vertical Jump

One of the simplest and generalized protocols when assessing the musculature of the lower limbs is the Bosco test [32]. For its recording, a contact mat was used which measures the vertical displacement [40]. Of all the jump tests proposed by Bosco, the counter movement jump, the same as in other studies [41], was the selected option. In this test, the individual, in an erect position and with the hands on the waist, carried out the maximum vertical jump after a downward counter-movement (flexion of the legs to 90°), maintaining the feet and knees in maximum extension from the lift-off up to the moment of reception.

To carry out the CMJ, a contact platform was used, “Ergo Jump Bosco System” [32] connected to a laptop unit (Ergo Tester Globus) which recorded the height of the jump (cm). All the subjects performed three jumps, recording the best one.

### 2.6. Hop Test/Unipodal Jump Test

The hop tests are monopodal and horizontal functional tests (lift-off and landing with the same leg) involving the values of strength, power, body control and coordination [33], which aim to imitate the demands of the knee’s dynamic stability [42].

These tests have been frequently used to identify the existence of asymmetries in the lower extremities, which can be quantified through the Limb Symmetry Index (LSI) [33], an accessible and valid indicator [42] when determining the dysfunction of one leg in relation to the other [43]. Asymmetry was calculated by dividing the lowest value by the largest and multiplying by 100.

Due to their frequency of use and their psychometric values, two unipodal jump tests were selected: single leg hop test and triple hop test [44,45]. The SLHT consists in the athlete’s execution of a unipodal hop as far as possible landing on the same leg without losing their control and balance [46]. Likewise, the THT [33] would assess the distance reached after the performance of three consecutive hops.

On an artificial turf surface where a start position was marked and a metric tape of 10 m of distance was placed adhered to the ground, the athletes, with proper sports footwear, performed three attempts with each leg in each of the tests, which were recorded for subsequent analysis of the best of the three attempts.

### 2.7. The 5 m Shuttle Run Sprint Test and Barrow Test/High Speed Changes of Direction

First due to its widespread use and high specificity in relation to the type of efforts which are required in soccer [47], we opted to perform the 5 m shuttle run sprint test. Demonstrated as valid and recommended by various authors [48,49], the test consists in a round trip route in a straight line repeated 10 times. Due to the preferences of the researcher team based on the criteria of lower risk for the subject, it was decided to modify the number of routes to eight.

Secondly, the barrow zigzag run test [34] was selected, since the same as the test above, it also measures the subject’s capacity to quickly change direction, turning, braking, accelerating and maintaining the overall control. The required equipment is minimal and includes a stopwatch and five cones laid out in a square of 5 × 5 m with a central cone which serves to signal the route to be completed. The psychometric properties of this test are optimal and recommendable [50].

The tests were explained and demonstrated to the participants. Prior to the performance of the attempts to be recorded, the athletes completed two familiarization trials. Due to the contextual limitations, a manual stopwatch was used in both tests [51]. To reduce the potential human error, the average value was recorded for the two attempts [52]. The recovery time between test and test was 3 min.

### 2.8. Data Analysis

For the injury analysis, we calculated the number of injuries and absence days per player.

For the study of the functional tests, a descriptive analysis was prepared for the values obtained in a collective and individual way during two seasons. By means of the Kolmogorov-Smirnov Test (K-S), the normality of each sample was established in each one of the tests. Subsequently, we carried out one-way ANOVA and Kruskall Wallis tests to establish differences between the groups according to the severity (absence days) and number of injuries.

When the intergroup differences were significant, we carried out post hoc Tukey tests and Mann Whitney U pair tests to specify the contrasts.

To establish a linear relation between the injuries of a player and their functional values, we applied the Pearson correlation coefficients (*r*) in the normal distributions and the Spearman Test (ρ) in the non-normal ones.

During the entire analysis process, we used the IBM SPSSS v.22 program with a significance level fixed at 0.05.

## 3. Results

A total of 125 injuries were recorded during the two seasons. Of these, 60 (48%) were minimal, 27 (21.6%) minor, 23 (18.4%) moderate and 15 (12%) severe. The majority of injuries were muscle/tendon (49.6%) and articular (25.6%) types. The total absence days amounted to 1432, which represents an average of 27.5 days/player.

For the total sample, the average of the three measurements in each of the tests was recorded in centimeters of height for the CMJ and in centimeters of difference for the Y balance directions. It was recorded in seconds for the barrow test and 8 × 5 m shuttle run, and the asymmetry percentage for the single and triple hop. The sample showed a non-normal distribution for the asymmetry variables in the unipodal jump (Table 1).

When dividing the sample based on the number of injuries suffered during the season, significant differences were only detected for the vertical jump test (CMJ). The players who did not suffer an injury were the ones who obtained the lowest values (Table 2).

When dividing the sample based on the number of absence days suffered, we again detected that the only difference occurred for the CMJ test and for the group of zero days (Table 3).

The correlation tests also did not show significant relationships between the value of a functional test and the number of injuries suffered or the number of absence days. None of the coefficients obtained a sufficient value to be considered as acceptable.

We can observe the dispersion and tendency line graphs for each of the tests in relation to the number of injuries suffered. Likewise, we can see this relation for the number of absence days.

## 4. Discussion

The aim of this study was to determine whether functional tests could detect the risk of injury during the season in professional soccer players. The functional tests have been traditionally used with three objectives: to determine the future performance of an athlete, to predict their injury risk and to control the progress of the injured player during his recovery process [18]. Despite numerous arguments in favor of their utility, the prediction capacity of the injury risk of these tests still lacks sufficient evidence [18,23,53].

Although several authors have found a correlation between the specific functional variables tested at the start of the season and the suffered injuries, the majority of the studies have been unable to demonstrate this relation [5,30,54]. Deficits of posture control [25,55,56], strength and asymmetry of lower limbs [25,45], as well as high speed and low aerobic capacity [57,58] have been several of the capacities which, in the absence of more evidence, seem to be best correlated with the risk of injury.

The data presented here corroborate the generalized feeling that they were unable to establish linear relations between the tests used and the number of injuries suffered and the number of absence days. When comparing the averages obtained by the groups based on the frequency and severity, we only detected that the players with lower jump strength (CMJ) had fewer injuries and had fewer absence days. The reduced number of subjects included in the groups with zero injuries and zero absence days (*n* = 5) hindered drawing conclusions in relation to this question.

The simple fact that a FPT presents a strong correlation between the number of injuries suffered is not a sufficient condition to guarantee the predictive power of this test [23]. This first step must be reinforced by establishing risk criteria based on the values obtained in the test to then determine if the players included in the risk groups suffer a higher number of injuries [23].

Finally, and as the main limitation of this study, it is important to remember that the explanatory models of the injury suggest that it is the result of a complex interaction among contexts, and intrinsic and extrinsic factors [2,59]. In view of this reality, the simplistic logic concerning what is sustained here when seeking the origin of an injury in a single factor (FPT) is not congruent with the complex and dynamic nature which the risk of suffering an injury possesses [60]. Multivariate studies which analyze the combination of different factors (subject-environment) and their prediction capacity must obtain a prominent role and be carried out in the near future [23].

## 5. Conclusions

- For the single hop, triple hop, Y balance, barrow and 8 × 5 m sprint tests, the values obtained by the soccer players who had from one to three injuries and from four to eight injuries during the season were similar to those obtained by the group with zero injuries suffered.
- For the single hop, triple hop, Y balance, barrow and 8 × 5 m sprint tests, the values obtained by the soccer players who were absent from one to 15 days, 16 to 28 days and more than 28 days during the season were similar to those obtained by the group that had no absence days.
- The players with zero injuries and zero absence days had lower values than the remainder in the CMJ test.
- There was no linear correlation among the functional values reached by the professional soccer players and the number of injuries and absence days suffered throughout the season.

## Figures and Tables

**Table 1:** Descriptive functional test and normality.

TEST	AVERAGE	NORMALITY (K-S)
**CMJ (cm)**	39.95 ± 4.16	0.20
**Barrow Test (s)**	7.46 ± 0.29	0.20
**8 × 5 m Shuttle Run Sprint Test (s)**	10.88 ± 0.39	0.20
**Asymmetry Single Hop (%)**	2.94 ± 1.75	0.00 *
**Asymmetry Triple Hop (%)**	2.39 ± 1.47	0.01 *
**YB—Right difference (cm)**	4.92 ± 1.72	0.93
**YB—Left difference (cm)**	3.52 ± 2.09	0.27

* *p* < 0.05 No normal distribution; CMJ: counter movement jump; s: seconds; cm: centimeters: YB: Y balance test.

**Table 2:** Descriptive functional test by number of injuries.

TEST	Uninjured (*n* = 5)	1–3 Injuries (*n* = 36)	4–8 Injuries (*n* = 11)	*p*
**CMJ (cm)**	35.56 ± 3.94	40.43 ± 4.42	40.36 ± 1.75	0.04 *
**Barrow Test (s)**	7.51 ± 0.28	7.47 ± 0.31	7.39 ± 0.22	0.68
**8 × 5 m Shuttle Run Sprint Test (s)**	10.96 ± 0.27	10.88 ± 0.43	10.87 ± 0.28	0.91
**Asymmetry Single Hop (%)**	3.35 ± 1.49	2.93 ± 1.82	2.78 ± 1.74	0.75
**Asymmetry Triple Hop (%)**	2.74 ± 1.40	2.40 ± 1.59	2.20 ± 1.12	0.65
**YB—Right difference (cm)**	5.25 ± 1.76	4.95 ± 1.92	4.72 ± 1.29	0.93
**YB—Left difference (cm)**	5.83 ± 0.23	3.36 ± 2.15	3.22 ± 1.98	0.27

* *p* < 0.05; CMJ: counter movement jump; s: seconds; cm: centimeters; YB: Y balance test.

**Table 3:** Descriptive functional test by number of absence days.

TEST	0 Days (*n* = 5)	1–15 Days (*n* = 24)	16–28 Days (*n* = 6)	+28 Days (*n* = 17)	*p*
**CMJ (cm)**	35.56 ± 3.94	39.62 ± 4.60	40.30 ± 4.00	41.58 ± 2.64	0.03 *
**Barrow Test (s)**	7.51 ± 0.28	7.47 ± 0.30	7.53 ± 0.36	7.39 ± 0.24	0.68
**8 × 5 m Shuttle Run Sprint Test (s)**	10.96 ± 0.27	10.95 ± 0.42	10.79 ± 0.49	10.80 ± 0.34	0.55
**Asymmetry SLHT (%)**	3.35 ± 1.49	3.94 ± 1.92	3.14 ± 1.59	2.60 ± 1.70	0.68
**Asymmetry THT (%)**	2.74 ± 1.40	2.55 ± 1.80	2.63 ± 1.06	1.99 ± 1.04	0.61
**YB—Right difference (cm)**	5.25 ± 1.76	5.57 ± 1.77	2.83 ± 1.25	4.64 ± 1.28	0.08
**YB—Left difference (cm)**	5.83 ± 0.23	3.75 ± 2.11	3.16 ± 1.75	2.75 ± 2.20	0.30

* *p* < 0.05; CMJ: counter movement jump; s: seconds; cm: centimeters; SLHT: single leg hop test; THT: triple hop Test; YB: Y balance test.
